# Comprehensive biomarker analyses identifies *HER2, EGFR, MET* RNA expression and thymidylate synthase 5'UTR SNP as predictors of benefit from S-1 adjuvant chemotherapy in Japanese patients with stage II/III gastric cancer

**DOI:** 10.7150/jca.34741

**Published:** 2019-08-28

**Authors:** Takaki Yoshikawa, Toru Aoyama, Kentaro Sakamaki, Takasi Oshima, Joyce Lin, Shenli Zhang, Nur Sabrina Sapari, Richie Soong, Iain Tan, Xiu Bin Chan, Dan Bottomley, Lindsay C Hewitt, Tomio Arai, Bin Tean Teh, David Epstein, Takashi Ogata, Yoichi Kameda, Yohei Miyagi, Akira Tsuburaya, Satoshi Morita, Heike I Grabsch, Patrick Tan

**Affiliations:** 1 Department of Gastric Surgery, National Cancer Center Hospital, Tokyo, Japan; 2 Department of Gastrointestinal Surgery, Kanagawa Cancer Center, Yokohama, Japan; 3 Department of Biostatistics and Epidemiology, Yokohama City University, Yokohama, Japan; 4 Cancer Therapeutics and Stratified Oncology, Genome Institute of Singapore, Singapore; 5 Cancer and Stem Cell Biology Program, Duke-NUS Graduate Medical School, Singapore; 6 Cancer Science Institute of Singapore, National University of Singapore, Singapore, Singapore; 7 Department of Pathology, National University of Singapore, Singapore, Singapore; 8 Cancer Therapeutics and Stratified Oncology, Genome Institute of Singapore, Singapore; 9 Division of Pathology and Data Analytics, Leeds Institute of Medical Research at St James's, University of Leeds, Leeds, UK; 10 Department of Pathology, GROW School for Oncology and Developmental Biology, Maastricht University Medical Center, Maastricht, NL; 11 Department of Pathology, Tokyo Metropolitan Geriatric Hospital and Institute of Gerontology, Tokyo, Japan; 12 Department of Pathology, Kanagawa Cancer Center, Yokohama, Japan; 13 Molecular Pathology and Genetics Division, Kanagawa Cancer Center Research Institute, Yokohama, Japan; 14 Department of Biomedical Statistics and Bioinformatics, Graduate School of Medicine, Kyoto University, Kyoto, Japan

## Abstract

**Purpose**: A comprehensive molecular analysis was conducted to identify prognostic and predictive markers for adjuvant S-1 chemotherapy in stage II/III Japanese gastric cancer (GC) patients and to evaluate their potential suitability for alternative cytotoxic or targeted drugs.

**Experimental Design**: We investigated genetic polymorphisms of enzymes potentially involved in 5-fluoruracil (5-FU) metabolism as well as platinum resistance, previously identified genomic subtypes potentially predicting 5-FU benefit, and mRNA expression levels of receptor tyrosine kinases and *KRAS* as potential treatment targets in a single institution cohort of 252 stage II/III GC patients treated with or without S-1 after D2 gastrectomy.

**Results**: 88% and 62% GC had a potentially 5-FU sensitive phenotype by SNP analyses of *TS* 3'UTR, and *TS* 5'UTR, respectively. 24%, 46%, 40%, 5%, and 44% GC had a potentially platinum sensitive phenotype by SNP analyses of *GSTP1, ERCC1* rs11615, *ERCC1* rs3212986, *ERCC2*, and *XRCC1*, respectively. High *HER2, EGFR, FGFR2*, or *MET* mRNA expression was observed in 49%, 66%, 72%, and 54% GC, respectively. High *HER2* expression was the only significant prognosticator (HR=3.912, 95%CI: 1.706-8.973, p=0.0005). High *HER2* (p=0.031), low *EGFR* (p=0.124), high *MET* (p=0.165) RNA expression, and *TS* 5'UTR subtype 2R/2R, 2R/3C, or 3C (p=0.058) were significant independent predictors for S-1 resistance.

**Conclusions**: The present study suggests that platinum-based or RTK targeted agents could be alternative treatment options for a substantial subgroup of Japanese GC patients currently treated with S-1. *HER2, EGFR, MET*, and *TS* 5'UTR SNP appear to be promising predictive markers for S-1 resistance warranting validation in an independent GC series.

## Introduction

Gastric cancer (GC) is the fourth most prevalent cancer and the third leading cause of cancer-related death worldwide 1. D2 gastrectomy is the mainstay of treatment for resectable GC in Japan 2, Europe and the US 3, 4. Two recent phase III trials in Eastern Asia demonstrated in patients with pathological stage II/III GC that adjuvant chemotherapy after D2 gastrectomy improved overall survival compared to surgery alone changing clinical practice in Japan 5, 6.

However, adjuvant S-1 chemotherapy does not seem to benefit all patients with stage II/III GC and severe or serious S-1 toxicity including anorexia, nausea, and diarrhea has been reported 5. Furthermore, 29% of ACTS-GC trial patients in the surgery plus adjuvant S-1 arm were not cured, whereas 61% of patients were cured by surgery alone 7 indicating that there is an urgent clinical need to (a) identify biomarkers that can better stratify patients for available treatment options than the currently used TNM stage after surgery and (b) to assess alternative treatment options such as other cytotoxic drugs (e.g. platinum) and/or targeted therapy.

Several previous GC studies have attempted to identify predictive or prognostic markers for S-1 chemotherapy. These studies included the investigation of protein expression of metabolic enzymes of 5-FU or folate such as thymidylate synthase (*TS*) 8-14, dihydropyrimidine dehydrogenase (*DPYD*) 8-10, orotate phosphoribosyl transferase (*OPRT*) 10-12, 14-16, thymidine phosphorylase 11, 13, or methylenetetrahydrofolate reductase 17, 18, the investigation of genetic polymorphisms of excision repair cross-complementing gene 1 (*ERCC1*) 17, 19, 20, or the investigation of expression of EGFR 11, 17, 21 or VEGF 22. However, all previous studies investigated usually only a small number of markers in each series and were often limited by the absence of a control group, small sample size, or use of a mixture of pre- and post-operative chemotherapy or resectable and unresectable disease. Furthermore, only two studies have investigated the relationship between biomarker and S-1 monotherapy 8, 21, while all other studies have used S-1 in combination with other cytotoxic drugs which may have influenced the results.

The aim of the current study was (a) to conduct a comprehensive molecular analysis of potential prognostic and predictive factors for adjuvant S-1 chemotherapy and (b) to evaluate the potential applicability of alternative cytotoxic or targeted drugs in a series of Japanese stage II/III gastric cancer patients treated with or without adjuvant S-1. Our analyses covered a wide range of biomarkers previously suggested to be related to benefit from S-1, 5-FU or platinum based chemotherapy, such as gene polymorphisms of *TS, OPRT, DPD, ERCC1, GSTP-1*, and to potential benefit from receptor tyrosine kinases (RTKs) targeting therapy.

## Patients and Methods

### Patients

This study was approved by the institutional review board of the Kanagawa Cancer Center, Yokohama, Japan. Gastric cancer patients were selected from a prospective database of the Kanagawa Cancer Center, Department of Gastrointestinal Surgery, Yokohama, Japan, according to the following criteria: (i) histologically proven gastric adenocarcinoma, (ii) curative gastrectomy as primary treatment between June 2002 and March 2010 following Japanese gastric cancer treatment guidelines published in 2010 2, (iii) pathologically stage II or III gastric cancer according to the 7^th^ edition of the TNM classification 23, and (iv) availability of at least 5 years follow up data from all patients.

**Figure 1:**
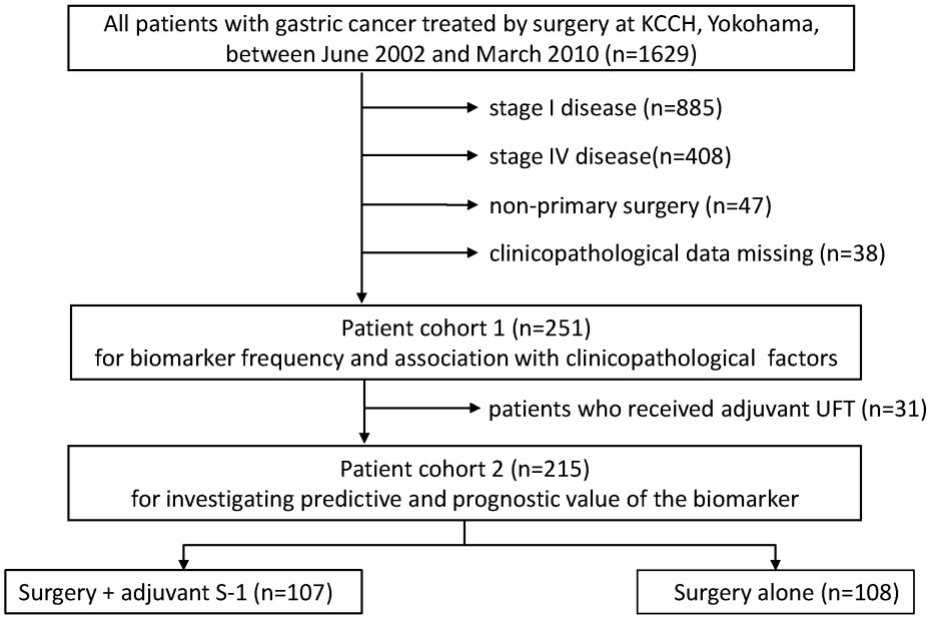
A flow diagram of patients involved in each analysis step

A total of 252 patients were selected for this study. A flow diagram of patients involved in each analysis step is presented in Figure 1. Associations with clinicopathologic factors were examined in all patients (cohort 1, n=252), while 22 patients who received UFT instead of S-1 as adjuvant chemotherapy and 15 patients who received palliative S-1 chemotherapy due to peritoneal cytology positive were excluded from survival analyses (cohort 2, n=215). Survival data were obtained from hospital records or from the Governmental registry system. The median follow-up period was 48.6 months (range 3.3-136.7 months).

### Preparation of the specimen and pathological diagnosis

Gastric cancer resection specimens were worked up according to the Japanese guidelines for gastric cancer 24. In addition, histological subtype according to the Lauren classification was determined. All slides from all resection specimens were reviewed by four senior gastrointestinal pathologists (HG, YM, TA, YK) to select and mark a representative block with the highest tumor cell density and a block with normal tissue for RNA and DNA extraction, respectively.

### DNA and RNA extraction

DNA for polymorphism analyses was extracted from normal tissue (non-metastatic lymph nodes or normal gastric wall) using the QIAamp DNA Micro kit (Qiagen, Hilden, Germany) and quality controlled as described previously 25. RNA was extracted from tumor tissue block sections after microdissection using the High Pure RNA Paraffin Kit (Roche, Mannheim, Germany) according to the instructions of the manufacturer. The extracted RNA was quantified by a Nanodrop UV-Vis Spectrophotometer (Thermo Fisher Scientific, Waltham, MA) and 100 ng was used for the Nanostring assay.

### Analyses of polymorphisms

#### *DPYD, OPRT, ERCC1, ERCC2* and *XRCC1* genotyping by MassARRAY

Genotype analysis was performed using the MassARRAY iPLEX system (Sequenom, San Diego, CA) according to manufacturer's instructions. A 7-plex assay interrogating *DPYD* (A1627G7&IVS14+1G>A), *OPRT* (G638C), *ERCC1* (C118T&C8092A), *ERCC2* (K751Q) and *XRCC1* (A399G) was designed using MassARRAY Online Design Tools (Sequenom) as described previously 26. Sample genotypes were determined by matrix-assisted laser desorption/ionization time-of-flight mass spectrometry (MALDI-TOF MS) analysis using the MassARRAY Compact system (Sequenom). The mass spectra analysis and genotype calls were generated using the Sequenom TYPER 4.0 software.

#### *GSTP1* and *TS* 3'UTR genotyping by Sanger sequencing

*GSTP1* (I105V) and *TS* 3'UTR (agttcat variant) genotyping was performed by Sanger sequencing. Purified pellets were dissolved in Hi-Di Formamide (Life Technologies) and analyzed using an ABI PRISM 3730 Genetic Analyzer (Life Technologies). Chromatograms were analyzed by SeqScape V2.5 (Life Technologies) and manual review.

#### *TS* 5'UTR genotyping by gel electrophoresis

*TS* 5'UTR were investigated as described previously 27. For *TS* 5'UTR, the predominant alleles expected at *TS* 5'UTR are the 2R and 3R alleles. PCR products from 2R and 3R alleles differ by a single 28 bp repeat, which can be resolved using gel electrophoresis. To determine the *TS* 5' UTR 2R/3R genotypes, 10 µl of PCR product was purified by 4 µl of Exonuclease I (New England Biolabs) and Thermosensitive Shrimp Alkaline Phosphatase (Promega) mixture treatment. After Exo-TSAP purification, PCR products were digested with 1 µl *HaeIII* (10,000 units/ml, New England Biolabs) followed by electrophoreses in 4% agarose/1xTBE gel.

### Nanostring Assay

A set of 203 genes selected based upon the molecular gastric cancer signature published by Tan *et al* 28 as well as other genes implicated in gastric carcinogenesis such EGFR, HER2, FGFR2, cMET and KRAS were selected for a customized Nanostring assay (Nanostring Technologies, Seattle, WA). The assay was performed according to the instructions of the manufacturer and the Nanostring workflow was employed for digital detection of expression counts for each gene. The 'NanoStringNorm' R package (version 2.15) was used to normalize the raw expression data adjusting for technical assay variations, background and RNA content. All samples flagged by the software were removed and the normalized expression data was used for all downstream analyses.

### Statistical analyses

Tumors were classified based on their expression signature as G-INT/G-DIF using the nearest template prediction algorithm as described previously 28. For the current study, we employed two different classification schemes. First, tumors were classified into two subtypes e.g. G-INT or G-DIF. Secondly, tumors were classified into 3 subtypes e.g. G-INT or G-DIF or G-ambiguous.

Associations between each biomarker and clinicopathological factors such as age, gender, location, tumor depth (pT), lymph node status (pN), distant metastasis (M), lymphatic channel invasion, blood vessel invasion, tumor size, macroscopic tumor type were examined in all patients. The analyses of the association between biomarker and histological subtype was restricted to those cases which were classified as either intestinal type or diffuse type GC. GC which were classified as mixed/unclassifiable according to Lauren (n=13) were excluded. Continuous factors (age, tumor size) were analyzed by student t-test, and categorical factors were analyzed by chi-square test. For analyses using distinct SNP subtypes, we excluded minor subtypes where the number of patients associated with a particular genotype (GG of GSTP1 in 5 patients and TT of ERCC1 rs3212986-C8092A in 10 patients) was felt to be too small for reliable statistical analysis. ERCC2 SNP was not analyzed for statistical analysis because most patients showed uniformal SNP type of TT (94.9%).

For survival analyses, the gene expression level cut off (high expression versus low expression) was determined by the maximum chi-square method 29. The validity of the selected cut off point was confirmed by a two-fold cross-validation approach for multivariate analysis 30. Overall survival probability curves were calculated using the Kaplan-Meier method and compared by the log-rank test. For the analyses of prognostic factors, a Cox's proportional hazard model was used to perform univariate and multivariate survival analyses in the surgery alone arm. A p-value of <0.05 was defined to be statistically significant for the analyses of the relationship of the biomarker with the clinicopathological factors including survival.

For the test of predictive factors, the interaction of each biomarker with S-1 treatment in the Cox's proportional hazard model was analyzed in the surgery alone and the surgery+S-1 arm. As this was a post hoc analysis that may not have enough power to detect an interaction effect, a raised significance level of p<0.2 was used to classify an interaction as being significant 31.

## Results

Table 1 shows the clinicopathological characteristics by treatment and cohort. Cohort 1 (all patients) was used for the analysis of the biomarker frequency and association with clinicopathological analyses, cohort 2 (excluding patients who received adjuvant chemotherapy other than S-1) was used for the prognostic and predictive analyses. As expected, patients in the surgery+S-1 group had cancers with higher pT category, higher TNM stage, larger tumor size, and were more frequent of scirrhous macroscopic tumor type (all p-values < 0.05). Although there was no statistically significant difference in overall survival (OS) between the surgery alone and surgery+S-1 group by log-rank test (p=0.177) in our series, the hazard ratio for surgery+S-1 chemotherapy was 0.694 (p=0.196) in multivariate analysis which was similar to that observed in the ACTS-GC trial 5.

### Frequency of biomarkers and association with clinicopathological factors

SNP genotypes suggested to predict 5-FU resistance e.g. *TS* 3'UTR (+6bp/-6bp and -6bp/-6bp) and *TS* 5'UTR (2R/3G, 3C/3G, and 3G/3G) were found in 88% and 62% GC, respectively. SNP phenotypes suggested to predict 5-FU toxicity e.g. *OPRT* (GC+CC) and *DPYD* (AG+GG) were present in 36% and 48% GC, respectively, while those suggested to predict platinum sensitivity e.g. *GSTP1* (AG+GG), *ERCC1* rs11615 (CT+TT), *ERCC1* rs3212986 (GT+TT), *ERCC2* variant (GT), and *XRCC1* rs25487 (AG+GG) were present in 24%, 46%, 40%, 5%, and 44% GC, respectively. High *HER2, EGFR, FGFR2*, or *MET* RNA expression identifying GC patients potentially benefitting from targeted therapy, was observed in 49%, 66%, 72%, and 54% GC, respectively.

*DPYD2A* was associated with the presence of distant metastasis (p=0.040). GT type of *ERCC1* and AA type of *XRCC1* were associated with distal location of the cancer (p=0.018) and female gender (p=0.043), respectively. The [+6bp/-6bp] type of *TS* 3'UTR and [2R/3C] or [3C/3C] type of *TS* 5'UTR were associated with younger age (p=0.022 and p=0.008, respectively). No other associations between individual SNP biomarker status and clinicopathological factors were seen.

Second, we examined associations between clinicopathological factors and gene expression, either at the single gene or gene signature level. High *EGFR* and high *FGFR2* RNA expression were associated with younger age (p=0.02 and p=0.01, respectively). High *EGFR*, low *HER2* and high *FGFR2* RNA expression were associated with diffuse type histology (p=0.001, p=0.009 and p=0.016, respectively). High *FGFR2* was associated with increased frequency of vascular invasion (p=0.047), high *MET* with distal location of the cancer (p=0.012) and low *KRAS* with presence of distant metastasis (p=0.022).

125 patients (51%) were classified as G-INT and 120 patients (49%) as G-DIFF when the two-category classification was employed. When the three-category classification was used, 88 (35.9%) patients were classified as G-INT, 71 (29%) patients as G-DIFF, and 86 (35.1%) patients as G-ambiguous. G-DIFF was associated with higher pT category (e.g. deeper invasion of the primary tumor) compared to G-INT or G-ambiguous type.

### Association of biomarker status and patient prognosis

**Figure 2:**
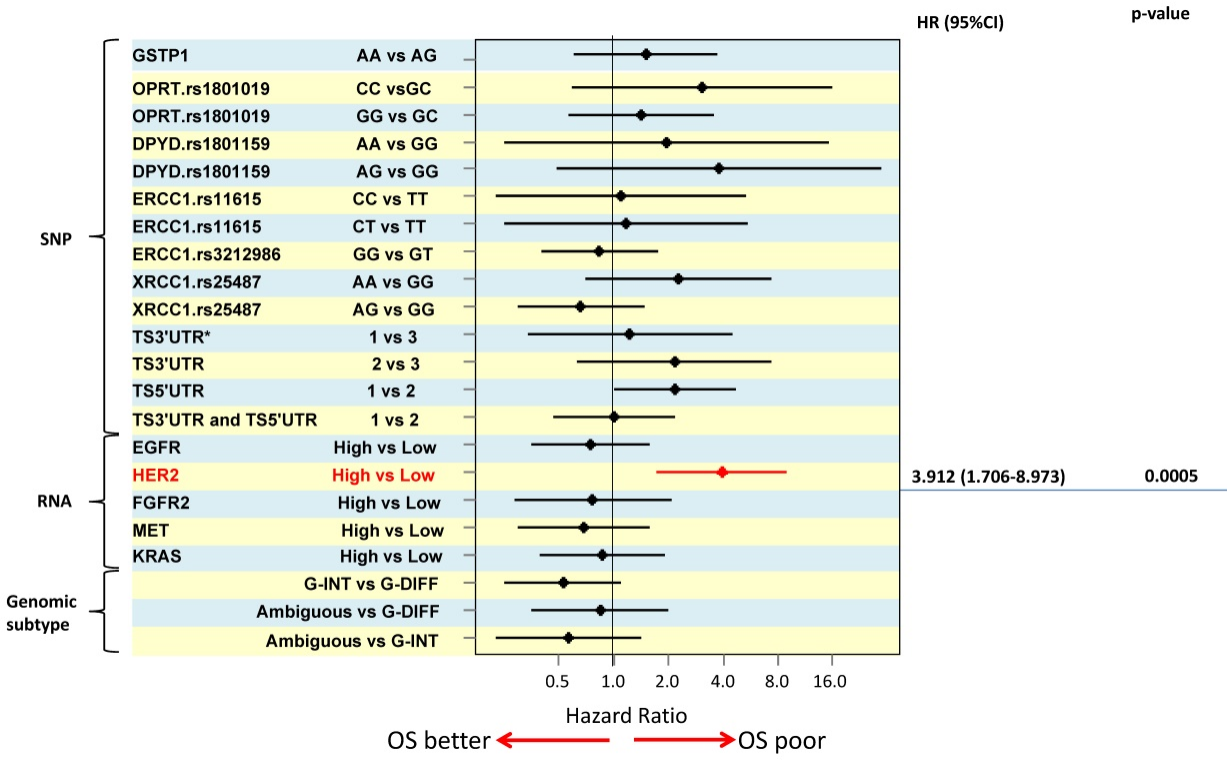
The results of the survival analyses are illustrated using a Forest plot of hazard ratios

In order to distinguish between predictive and prognostic value of a biomarker, associations with survival were analysed separately by treatment e.g. the prognostic value of a marker was determined using data from the surgery alone treated patient group. For multivariate overall survival analyses, TNM stage, tumor size and macroscopic tumor type were included in the model together with the biomarker of interest. The results of the survival analyses are illustrated using a Forest plot of hazard ratios (Figure 2). High *HER2* RNA expression was associated with poor survival and was the only significant independent prognosticator (HR=3.912, p=0.0005). Patients with *TS* 5'UTR 2R/3G or 3C/3G or 3G/3G tended to have poorer survival than those with 2R/2R, 2R/3C, or 3C/3C (HR =2.185, p=0.058) but the difference did not reach statistical significance.

### Association of biomarker status and patient benefit from adjuvant S-1 chemotherapy

**Figure 3:**
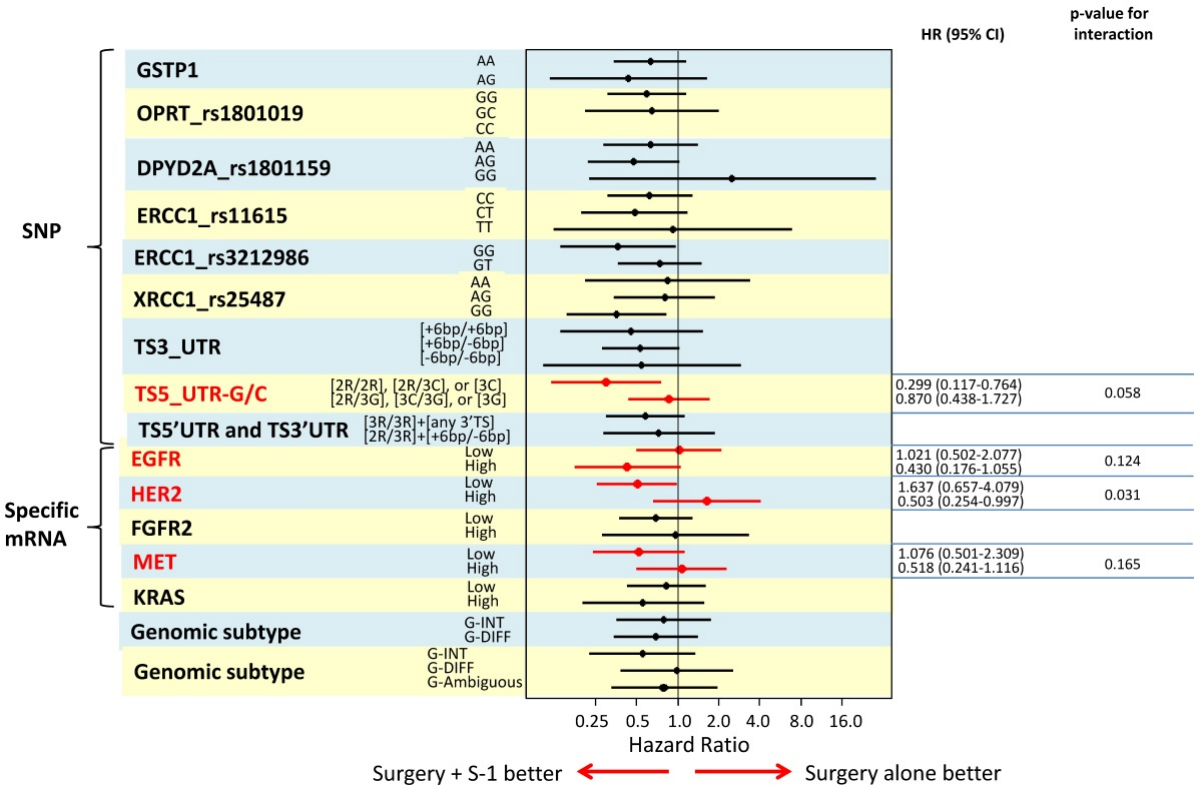
The results are illustrated in a Forest plot of hazard ratios

To identify biomarkers that might predict which GC patient subgroup will benefit from adjuvant S-1 therapy, we determined the interaction of each biomarker with S-1 treatment using a Cox's proportional model by including S-1 treatment, TNM stage, tumor size, macroscopical tumor type, and biomarker in the model. The results are illustrated in a Forest plot of hazard ratios (Figure 3). We found that *TS* 5'UTR subtype 2R/2R, 2R/3C, or 3C/3C (p=0.058), high RNA expression of *HER2* (p=0.031), low *EGFR* expression (p=0.124) and high *MET* expression (p=0.165), were significant independent predictors for S-1 resistance.

## Discussion

The present study reports our comprehensive analysis of prognostic and predictive biomarkers in a cohort of Japanese gastric cancer treated by surgery with and without adjuvant S-1 chemotherapy. S-1 is a drug which consists of tegafur, a prodrug of 5-FU, gimeracil, a competitive inhibitor of DPYD, and oteracil potassium, an inhibitor of 5-FU phosphorylation. We explored a number of different biomarkers including (i) genetic polymorphisms of enzymes involved in 5-FU metabolism such as *OPRT* 15, *DPYD* 32, and *TS* 33, (ii) genetic polymorphisms of proposed predictors for platinum resistance 34, 35 such as *GSTP-1*, *ERCC1*, and *XRCC1*, (iii) previously identified GC genomic subtypes (G-INT and G-DIFF 28) as potential prognosticators as well as predictors for 5-FU sensitivity and (iv) RNA expression of *EGFR, HER2, FGFR2, MET*, and *KRAS* to evaluate the potential for therapies targeting the RTK/KRAS signaling pathway in Japanese gastric cancer currently treated with surgery plus S-1 chemotherapy.

Our study showed no relationship between *OPRT* and *DPYD* SNPs and S-1 treatment benefit, only the *TS* 5'UTR SNP predicted benefit from S-1 adjuvant chemotherapy. This is in contrast to previous studies which suggested that protein, mRNA, or genetic polymorphism of TS 8-14, DPYD 8-10, and OPRT 10-12, 14-16 were predictors of response to 5-FU based chemotherapy. However, most previous studies did not have a non-chemotherapy control arm and thus were not able to distinguish between prognostic and predictive value of a biomarker or suffered from relatively small sample size. Our study is the first to clarify the predictive and prognostic impact of these enzyme SNPs on S-1.

TS is an enzyme which catalyzes the methylation of fluoro-dUMP to dTMP, an essential precursor for DNA synthesis 36. 5-FU inhibits TS activity and blocks DNA synthesis 18. It is thus biologically plausible that S-1 (a 5-FU prodrug) efficacy might also be influenced by TS activity. The promoter enhancer region of *TS* gene is polymorphic, containing a double (2R) or triple (3R) tandem repeat known to be involved in the translational auto-regulation mechanism of TS expression 37. Previous investigators reported that 2R/2R, 2R/3C, and 3C/3C type of *TS* polymorphisms were related with better survival in colorectal and gastric cancer patients receiving fluoropyrimidine 38. However, their study did not include a surgery alone group and thus cannot distinguish between prognostic and predictive value of the SNP. There is currently no study published investigating a relationship between *TS* polymorphism and S-1 efficacy. The present study was able to clarify that *TS* 5'UTR polymorphisms have no prognostic value in Japanese gastric cancer patients (e.g. do not influence the survival in the surgery alone group) but does predict benefit from adjuvant S-1 treatment depending on the SNP subtype of *TS* 5'UTR. This finding, if replicated in a second independent series, could indicate that determination of the *TS* 5'UTR SNP status which would be possible in any normal tissue including a routine blood sample might be a useful marker to stratify patients for adjuvant S-1 therapy and/or other therapy options.

It has been shown recently that classifying gastric cancer based on their RNA expression signature into G-INT and G-DIFF identifies groups of patients with different prognosis 28. Moreover, the genomic subtype seemed to predict benefit from adjuvant 5-FU based chemotherapy 28. However, when using the same classifier in the present study, the genomic classifier did neither predict patient prognosis nor benefit from adjuvant S-1. This discrepancy could be related to the stage selection bias in the current study which included only stage II/III gastric cancer patients whereas the previous study included all disease stages. Secondly, differences in drug efficacy between S-1 and 'conventional 5-FU' have been described for diffuse type gastric cancer 39 and might explain the different results.

In addition to the investigations into prognostic and predictive marker for S-1 benefit, we also used this gastric cancer cohort to explore whether there is any indication that patients might benefit from potential other cytotoxic drugs such as platinum based chemotherapy. Polymorphisms for *GSTP-1*, *ERCC1* and *XRCC1* have been previously identified as potential predictive biomarker for platinum based chemotherapy in colon cancer patients 34, 35, 40, 41. *ERCC1* SNPs have shown to have prognostic and/or predictive value in the advanced (metastatic) gastric cancer setting 17, 19, 35, 42-44. In the current series, SNPs of *GSTP-1, ERCC1*, and *XRCC1* were neither a prognosticator nor a predictor for S-1 adjuvant chemotherapy suggesting that adding platinum drugs to S-1 might improve patient's prognosis.

Similarly, we explored whether patients in our series would potentially be eligible for RTK targeting drug therapy such as HER2 targeting trastuzumab, pertuzumab or T-DM1; EGFR targeting nimotuzumab, FGFR2 targeting AZD4547 or MET targeting rilotumumab or onartuzumab or MEK inhibitors which may be attractive therapies for KRAS-driven tumors. The present study demonstrated that high *HER2* RNA expression was associated with poor prognosis and low *EGFR*, high *HER2* and high *MET* RNA expression were important predictor for S-1 resistance.

Our data seem to indicate that whereas therapy targeting HER2 or MET should not be combined with S-1 chemotherapy, a combination of EGFR targeting drugs with S-1 chemotherapy might be of added value. However, a recent study in a subset of patients from the ACTS-GC trial 5 reported that HER2 'positivity' by immunohistochemistry or copy number analyses was not a significant prognosticator and that neither EGFR nor HER2 status predicted benefit from S-1. The results from this study are not directly comparable with the current study as different methodology (RNA expression level in the current study versus protein expression and DNA copy number in the previous study) was used to establish the HER2/EGFR status of the individual case. Ma *et al* reported a low correlation between HER2 protein expression and RNA expression and suggested that HER2 protein and RNA may have a different clinical role in gastric cancer patients 45. On the other hand, there is currently no information available about the relationship between RNA and protein expression levels in EGFR in gastric cancer.

The present study has some limitations. This is a retrospective, single center case-control study with no treatment randomization. Thus, in contrast to the results from the ACTS-GC trial, the univariate overall survival curves were similar between the S-1 group and surgery alone group in the current study. However, the hazard ratio was almost identical to that observed in S-1 arm of ACTS phase III study when the survival was adjusted by Cox's multivariate analysis. In addition, the present study was only analyzed the Eastern cohort. Therefore, the further study should focus on the Western cohort. Furthermore, our study results require validation in a second independent cohort of gastric cancer patients treated with adjuvant S-1 which was not achievable within the current study cohort due to its sample size.

In conclusion, this is the first comprehensive biomarker analysis comparing biomarker status in stage II/III gastric cancer patients treated with surgery alone to those treated with surgery plus adjuvant S-1 chemotherapy. We identified several candidate markers such as *HER2, EGFR, MET* RNA expression and *TS* 5'UTR SNP which are able to identify patients with a high risk of recurrence and/or no potential benefit from adjuvant S-1 therapy. The findings of this study may potentially inform future trial design to test platinum drugs and targeting agents after biomarker stratification in order to improve survival of Japanese stage II/III gastric cancer. The promising results from this comprehensive but exploratory biomarker analyses require validation in a second series before clinical application.

## Figures and Tables

**Table 1:** Clinicopathological characteristics of patient cohort 1 (n=252) and patient cohort 2 (n=215; excluding 22 patients treated with UFT adjuvant chemotherapy and 15 patients who had positive peritoneal cytology and received palliative S-1 chemotherapy (*))

		Surgery alone		Surgery + S-1	
		n	%	n / n*	%/ %*
Age	Median (range) years	66 (35-85)		64 (35-83) 64 (35-80)*	
Gender	Male	74	68.5	102 / 73	70.8 / 68.2
	Female	34	31.5	42 / 34	29.2 / 31.8
Tumor location	Upper third	29	26.9	47 / 35	32.6 / 32.7
	Middle third	41	38	62 / 48	43.1 / 44.9
	Lower third	38	35.2	35 / 24	24.3 / 22.4
Tumor size	Median (range) mm	50 (15-180)		60 (25-212) 60 (28-212)*	
Macroscopic tumour type	0	25	23.1	14 / 10	9.7 / 9.3
	1	6	5.6	7 / 6	4.9 / 5.6
	2	26	23.1	36 / 26	25.0 / 24.3
	3	28	26.9	37 / 24	25.6 / 22.4
	4	2	1.9	18 / 16	12.5 / 15
	5	21	19.4	32 / 25	22.3 / 23.4
Depth of invasion (pT)	T1/T2	31	28.7	18 / 16	12.5 / 15
	T3/T4	77	71.3	126 / 91	87.5 / 85
Lymph node status (pN)	N0	20	18.5	22 / 19	15.3 / 17.8
	N1/N2/N3	88	81.5	122 / 88	84.7 / 82.2
TNM stage	2A	18	16.7	8 / 5	5.6 / 4.7
	2B	36	33.3	36 / 31	25.0 / 29
	3A	25	23.1	18 / 11	12.5 / 11.2
	3B	14	13	29 / 25	20.1 / 22.4
	3C	15	13.9	53 / 35	36.8 / 32.7
Lymphatic invasion	negative	42	38.9	45 / 39	31.3 / 36.4
	positive	66	61.1	99/ 68	68.7 / 63.6
Venous invasion	negative	29	26.9	34 / 32	23.6 / 29.9
	positive	79	73.1	110 / 75	76.4 / 70.1
Histological tumor type	Intestinal	40	37.4	42 / 34	29.1 / 31.8
	Diffuse	62	57.4	95 / 67	66.0 / 62.6
	unclassifiable	6	5.5	7 / 6	4.9 / 5.6
